# Logical modelling reveals the PDC-PDK interaction as the regulatory switch driving metabolic flexibility at the cellular level

**DOI:** 10.1186/s12263-019-0647-5

**Published:** 2019-09-09

**Authors:** Samar HK Tareen, Martina Kutmon, Ilja CW Arts, Theo M de Kok, Chris T Evelo, Michiel E Adriaens

**Affiliations:** 1 Maastricht Centre for Systems Biology (MaCSBio), Maastricht University, Maastricht, The Netherlands; 2 Department of Bioinformatics – BiGCaT, NUTRIM School of Nutrition and Translational Research in Metabolism, Maastricht University, Maastricht, The Netherlands; 3 Department of Epidemiology, CARIM School for Cardiovascular Diseases, Maastricht University, Maastricht, The Netherlands; 4 Department of Toxicogenomics, GROW School of Oncology and Developmental Biology, Maastricht University, Maastricht, The Netherlands

**Keywords:** Metabolic flexibility, PDC, PDK, Logical modelling, Metabolism, Regulation, Regulatory network, Glycolysis, Fatty acid oxidation

## Abstract

**Background:**

Metabolic flexibility is the ability of an organism to switch between substrates for energy metabolism, in response to the changing nutritional state and needs of the organism. On the cellular level, metabolic flexibility revolves around the tricarboxylic acid cycle by switching acetyl coenzyme A production from glucose to fatty acids and vice versa. In this study, we modelled cellular metabolic flexibility by constructing a logical model connecting glycolysis, fatty acid oxidation, fatty acid synthesis and the tricarboxylic acid cycle, and then using network analysis to study the behaviours of the model.

**Results:**

We observed that the substrate switching usually occurs through the inhibition of pyruvate dehydrogenase complex (PDC) by pyruvate dehydrogenase kinases (PDK), which moves the metabolism from glycolysis to fatty acid oxidation. Furthermore, we were able to verify four different regulatory models of PDK to contain known biological observations, leading to the biological plausibility of all four models across different cells and conditions.

**Conclusion:**

These results suggest that the cellular metabolic flexibility depends upon the PDC-PDK regulatory interaction as a key regulatory switch for changing metabolic substrates.

**Electronic supplementary material:**

The online version of this article (10.1186/s12263-019-0647-5) contains supplementary material, which is available to authorized users.

## Introduction

Metabolic flexibility is the ability of an organism to switch between substrates for energy metabolism, adapting to the changing nutritional state and needs of the organism [1]. In complex organisms, such as humans, the various cells and tissues utilising either glucose or fatty acids and its derivatives to fuel metabolism maintain metabolic flexibility. This flexibility revolves around the tricarboxylic acid (TCA) cycle in oxidative metabolism, where several biochemical processes interact with each other to use either glycolysis or fatty acid oxidation to fuel metabolism. Due to various tissues and organs having different energy requirements, metabolic flexibility in complex organisms also includes the delicate balance between these tissues and organs in utilising the correct substrate at correct times, so as not to starve off limited supply of nutrients critical to the functioning of other organs and tissues [1]. This cellular and tissue/organ level metabolic flexibility works collectively to manage the nutrient state and needs of the organism, and by design enforces the utilisation of a single substrate on the cellular level. Recent studies have found impaired metabolic flexibility to be associated with obesity and related co-morbidities, chiefly type 2 diabetes mellitus and cardiovascular diseases [2–4].

In a recent review [5], we explored the various cellular processes involved in maintaining cellular metabolic flexibility in the adipose tissue. The two major energy production mechanisms, glycolysis and fatty acid oxidation, are tied to the TCA cycle – glycolysis through the production of pyruvate and its conversion to acetyl coenzyme A (acetyl-CoA), and fatty acids through their breakdown to acyl coenzyme A and transportation into the mitochondria through the carnitine transport mechanism for eventual conversion to acetyl-CoA. This acetyl-CoA is converted to citrate, which starts the TCA cycle converting adenosine monophosphate (AMP) to adenosine triphosphate (ATP) and oxidised nicotinamide adenine dinucleotide (NAD+) to its reduced form NADH, improving the energy state of the cell. Excess energy, in the form of citrate escapes to the cytoplasm from the mitochondria, where it shuts down glycolysis and/or fatty acid oxidation and contributes to the re-synthesis of cellular fatty acids. Some additional cellular processes also assist in the regulation of cellular metabolic flexibility, namely the adenosine monophosphate-activated protein kinase (AMPK) signalling cascade and the peroxisome proliferator-activated receptor gamma (PPAR *γ*) nuclear receptor mediated transcriptional regulation [5].

In another study of ours [6], we used published data to generate clusters of correlated genes preserved in the majority of individuals that participated in a weight loss study [7]. We observed that one of the generated clusters was primarily involved with the upstream regulation of the TCA cycle. This observation suggests additional links between the regulation of cellular metabolic flexibility and obesity related co-morbidities, considering that weight loss is the predominant method of countering obesity and its ill effects. In our review [5], we also highlighted an inhibitory regulatory interaction directed from pyruvate dehydrogenase kinase (PDK) to pyruvate dehydrogenase complex (PDC) as a key nutrient switching mechanism between glucose and fatty acids, especially since we observed it to be affected in obesity.

Based on these observations, we hypothesise that this regulatory interaction, termed by us as the PDC-PDK regulatory switch, is a key regulator of cellular metabolic flexibility. As such, we focused on the changing of the metabolic substrate in response to the PDC-PDK regulatory interaction in this study. We have used logical modelling (i.e., a predicate logic based modelling framework) to construct a regulatory model to test this hypothesis, and show how the various perturbations designed to derail cellular metabolic flexibility are propagated through the malfunctioning of the PDC-PDK regulatory switch. We opted to use logical modelling, as opposed to more complex and complicated quantitative modelling primarily because of the high resolution of data required to accurately model cellular processes quantitatively [8]. Molecular interactions in the cell, such as protein associations, occur rapidly at rates from less than 10^3^ M ^−1^*s*^−1^ to greater than 10^9^ M ^−1^*s*^−1^ [9] and require specialised experiments and lengthy simulations to determine accurately [10]. Many of the cellular interactions that we modelled in this study currently have little-to-no accurate in vivo measurements available on smaller time scales at which they occur to properly construct and train a quantitative regulatory model. These interactions include, but are not limited to, protein-protein interactions, site-specific phosphorylation, allosteric interactions, chemical associations and dissociations. As logical modelling does not rely on material and spatiotemporal quantification of entities, it has been successfully applied previously in scenarios with sparse data availability [8, 11–13].

## Materials and methods

**Fig. 1:**
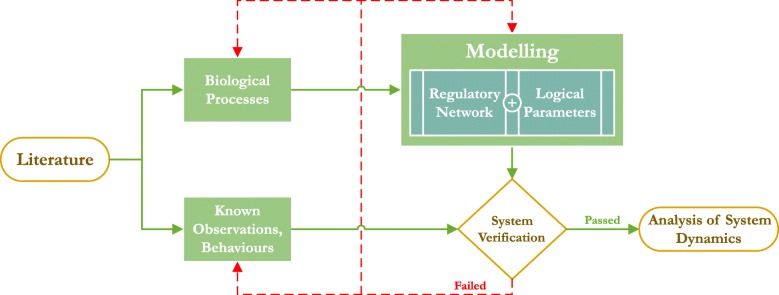
Workflow of the methodology. Biological processes and their known observations were extracted from literature. These processes were then used to construct the regulatory network with the logical parameters selected based on the biochemical reactions and known interactions. The regulatory network then underwent system verification where it was tested to check if it could exhibit known biological observations and behaviours, or not. If the verification failed, then troubleshooting was performed by checking the model for errors, changing the system definitions extracted from the literature, and/or checking if the known observations were in conflict with the system. If the system verification passed, then the dynamics generated by the model were analysed for biologically meaningful behaviours

We applied a well-established pipeline of logical modelling and analysis of biological pathways and networks using discrete/qualitative models [11, 14–16]. The pipeline started with the construction of the biological regulatory network, after which its parameters were defined via logic circuits (which can also be represented in a tabulated manner). Collectively, the network and the parameters constitute a single model, with multiple parameter sets representing distinct models of the same regulatory network. Using the parameters, a new network called a state transition graph (STG) was constructed, representing all possible behaviours of the regulatory network in the discrete state space. This network was then further analysed for relevant biological behaviours, both for system verification and for predictions. Figure 1 represents the overview of this methodology, and we have used a toy example to illustrate the procedure step-by-step in the subsequent sections.

### Logical modelling

In logical modelling, the model represents a system by using discrete values of 0 and 1 for OFF and ON states of the entities comprising the system. The dynamics of the system are then defined by step functions that change these values. In our study, we have employed the René Thomas Kinetic Logic formalism [17] and refer to the work of Paracha et al. [14] for the mathematical definitions and constraints of the formalism. Of note is the distinction that we defined 1 as availability and 0 as unavailability of an entity in this study. This availability and unavailability, however, does not imply any concentration of the said entity, only whether the entity is able to perform its functions or not. We used this interpretation primarily to model allosteric inhibitory interactions which are otherwise difficult to model in a concentration-based interpretation (since the concentration-based interpretation would imply that the allosteric inhibition and allosteric activation always decreases and increases the production of the target entity respectively, which may not always be the case in reality). In the following sections, we show the modelling and analysis of a toy example to ease the reader into the application of the formalism.

#### Regulatory network

**Fig. 2:**
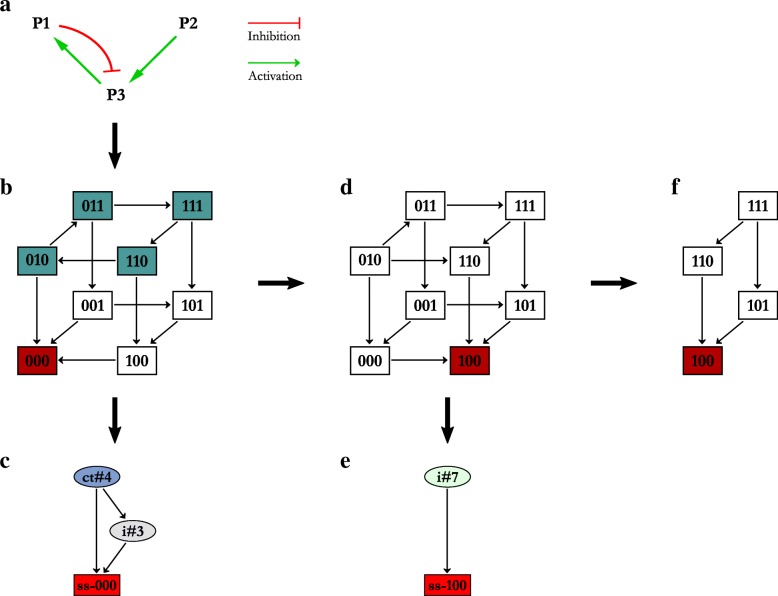
Step-by-step analysis of the toy example. **a**) The regulatory network of the toy example. P1 and P2 activate P3, whereas P1 inhibits P3. **b**) The state transition graph (STG) of the toy example. **c**) The hierarchical transition graph (HTG) of the toy example. **d**) The STG of the toy example with P1 showing ectopic activity. **e**) The HTG of P1 ectopic activity. **f**) The STG of P1 ectopic activity when the system is initialised with all entities as active (i.e. at level 1). The STGs and HTGs were generated using GINsim [19]

In the toy example, we have a regulatory network consisting of three entities, P1, P2 and P3. P1 and P3 have a reciprocal relationship where P3 is an activator of P1, and P1 the inhibitor of P3. An activator implies that the source entity has a positive effect on the concentration and/or activity of the target entity, whereas an inhibitor has a negative effect. We also see P2 as an activator of P3, and by extension, having an indirect effect on the activity of P1 mediated via P3. The regulatory network for the toy example is shown in Fig. 2a. One constraint in the René Thomas formalism is that the maximum discrete level attainable by an entity is constrained by the total number of its target entities [17]. In the toy example, all three entities have a single target entity, and are thus constrained to a maximum discrete level of 1 each.

#### Logical parameters

As multiple entities can affect the same target entity simultaneously, a set of logical parameters was selected which define the rules and precedence governing the evolution of the target entities in the network. These logical parameters are based on the behaviours observed, measured or inferred using data and experiments. In the toy example, we define the logical parameters based on the interactions that we have in the regulatory network,

1. P2 is the activator of P3.
2. P3 is the activator of P1.
3. P1 is the inhibitor of P3.

We see that the logical parameters for P1 are trivial as it has only one activator and no inhibitor. Generally, in the absence of their activators, the entities are assumed to degrade over time. Thus, P1 would be reduced to level 0 in the absence of P3, whereas P2 would reduce to 0 as its activator has not been modelled in the regulatory network. It is also possible to assume an implied activator that would activate P2, but generally, such assumptions are made only if an inhibitor of the entity in question has been explicitly modelled in the regulatory network to balance the activity of the said entity. Since we do not have any such an inhibitor for P2 in the regulatory network, we assume otherwise. Lastly, the logical parameters for P3 are non-trivial since we have both an activator (P2) and an inhibitor (P1) that can act simultaneously on P3. Here we model a precedence for the inhibitor, and assume that P3 would always be inhibited by P1 whenever P1 is present in the system, otherwise P3 would rely on P2 to become activated. These parameters are tabulated as Table 1.

**Table 1:** Logical parameters of the toy example

Entity	Parameter Set	Target Value
P1	{ }	0
	{P3}	1
P2	{ }	0
P3	{ }	0
	{P1}	0
	{P2}	1
	{P1, P2}	0

The format used here represents the presence of respective entities in the system when they are listed in the parameter set

#### State transition graphs (STGs)

The logical parameters govern the behaviour of the regulatory network, from which it is possible to generate a graph of all possible behaviours. This graph is called a State Transition Graph (STG), consisting of states (nodes) and transitions (edges). Each state represents a particular configuration of the complete regulatory network, where a configuration is defined as a unique combination of the discrete levels of the entities of the regulatory network. Thus, any two states would have a different discrete level for at least one entity. The maximum number of states is defined by the formula *lvl*^*n*^, where *lvl* represents the maximum discrete level plus 1 (essentially the total number of levels available for a given entity), and *n* represents the number of entities having that level. In the toy example, this formula would be 2^3^, totalling eight states, as we can see in Fig. 2b.

The transitions represent the changes in the discrete levels of the entities, and thus the transitioning of the regulatory network from one configuration to another. The transitions are constrained by the logical parameters, and can only exist between two states if the source state satisfies the discrete levels of the target state through the logical parameters. This imparts directionality to the behaviours represented in the STG, generating cyclic and acyclic paths in the graph. A behaviour can then be defined as a path in the STG, essentially a series of states connected by transitions between them. Finally, we utilise asynchronous transitions, which only allow one entity to change its discrete level between two successive states. The logical parameters given in Table 1 were used to derive the STG of the toy example shown in Fig. 2b. Each state is labelled with three numbers, representing the discrete levels of P1, P2, and P3 in that order (for example, state 101 refers to P1 and P3 having level 1, and P2 having level 0).

### System verification

Using the STGs generated by a regulatory network, it is possible to reverse engineer sets of logical parameters that allow certain known behaviours of the system to exist in the regulatory network. By extension, this allowed our modelled system to be verified against known biological observations or to find logical parameters that satisfy those conditions. We utilised a model checking technique called computational tree logic (CTL) to identify known behaviours [18]. CTL allowed us to use predicate logic along with quantifiers to formulate behaviours, and test which sets of logical parameters allow such behaviours to exists within their STGs. Specifically, for a given predicate formula *ϕ*, these quantifiers are;

- AG *ϕ*: From a given state, all states (G) along all paths (A) must satisfy *ϕ*
- AF *ϕ*: From a given state, at least one future state (F) along all paths (A) must satisfy *ϕ*
- EG *ϕ*: From a given state, all states (G) along at least one path (E) must satisfy *ϕ*
- EF *ϕ*: From a given state, at least one future state (F) along at least one path (E) must satisfy *ϕ*
- AX *ϕ*: From a given state, all (A) immediate successor states (X) must satisfy *ϕ*
- EX *ϕ*: From a given state, at least one (E) successor state (X) must satisfy *ϕ*

For the toy example, we formulate the CTL formula as, ((*P*2=1) → *EX*(*EG*(*P*2=1))) ∧ ((*P*2=1) → *EX*(*EG*(*P*2=0)))

The formula states that when P2 is at discrete level 1, there exists at least one path from at least one successor state, which maintains P2 at level 1, AND there exists at least one path from at least one successor state which reduces the level of P2 to 0 indefinitely. When applied, this formula is tested against all STGs produced by all possible logical parameter sets for the toy example regulatory network. Only 2 logical parameter sets out of the total 36 are able to satisfy this property, one of which is already given as Table 1. Additional file 1 provides the source file for the toy example system verification, and includes the definition of the network, the logical parameters, and the CTL formula.

### Network analysis

Once the system was verified, we proceeded with the analysis of the behaviours provided in the STG. The results of such an analysis for the toy example is visualized in Fig. 2. The blue shaded states represent a cyclic behaviour where the system can keep transitioning from one state after the other, successively and indefinitely. We can also see that the entity P2 maintains its level 1 as long as the system remains within this cyclic behaviour. As soon as P2 changes its level to 0, the system transitions away from the cyclic behaviour into several separate acyclic behaviours, all of which led the system to a deadlock state 000. A deadlock state, also referred to as a stable steady state or fixed point, is defined as a state in an STG, which has no exit transitions, and implies that the system gets stuck in this state. The acyclic states are coloured white, whereas the deadlock state is coloured red in Fig. 2b. Cyclic behaviours/trajectories represent periodic or recurring biological processes, such as circadian rhythms, while acyclic behaviour/trajectories represent one-way propagations, such as signalling cascades. In Fig. 2b, we can see that the maintenance of P2 at level 1 is required to keep the system in a periodic behaviour.

#### Hierarchical transition graphs (HTGs)

One of the drawbacks of logical modelling is state-space explosion – the size of the STG increases exponentially with linear increase in the size of the regulatory network. For example, increasing our regulatory network to four entities would yield an STG of sixteen states, while six entities would create an STG of sixty-four states. Subsequently, the STG of larger regulatory networks becomes very complex, and extremely tedious and error-prone for manual analysis. However, it is possible to analyse large STGs by finding sub-networks and patterns (such as strongly connected components (SCCs), essentially linked cyclic paths) contained within the STG itself. Towards this end, GINsim allows us to collapse these sub-networks and patterns in the network to generate Hierarchical Transition Graphs (HTGs) [19–21]. The collapsed substructures as then represented as,

- Transient SCC: a node containing a strongly connected component, which also has outgoing transitions to other components or parts in the HTG. These nodes are labelled as ‘ct#’ followed by the number of states contained within, e.g., ‘ct#4’ in Fig. 2c.
- Terminal SCC: a node containing a strongly connected component, which does not have any outgoing transitions to other components. These nodes are labelled as ‘ca#’ followed by the number of states.
- Irreversible Component: a node containing states and transitions that do not have any cycles in them. Such components represent unidirectional flow in the behaviours being represented by the HTG. These nodes are labelled as ‘i#’ followed by the number of states.
- Rooted Irreversible Component: an irreversible component that includes at least one state with no incoming transitions. These nodes are labelled the same as irreversible components, i.e., ‘i#’ followed by the number of states.
- Stable State: a node containing a single deadlock state which the system is unable to exit upon entering i.e., it has no outgoing transitions. These nodes are labelled as ‘ss-’ followed by the label of the state/configuration itself. For example, the nodes ‘ss-000’ and ‘ss-100’ in Fig. 2c and 2d respectively.

We then proceeded to collapse the acyclic paths into single nodes, and finally merged all edges between these collapsed nodes based on the edges present between the respective states in the STG. Figure 2c shows the HTG for the toy example, where the cyclic path has been collapsed to the light blue node ‘ct#4’, and the acyclic path has been collapsed to the grey node ‘i#3’, with the deadlock state represented as its own node ‘ss-000’. Thus, the HTG provides a structural representation of the system by illustrating the connections between various sets of behaviours found in the underlying STG. In addition, HTGs are by definition acyclic due to the collapsing procedure.

#### Perturbation analysis

In addition to the HTG, we also performed the perturbation analysis, where we restricted the parameters of one or more entities to represent knockouts or ectopic activities. The STG constructed with these restrictions establishes the propagation of their effects through the rest of the system. Figure 2d shows the STG when entity P1 is restricted to ectopic activity. We can immediately see that the cyclic behaviour found in the STG in Fig. 2b is no longer available, and that the deadlock state has moved from 000 to 100. In addition, its HTG (Fig. 2e) shows only two nodes, a rooted ’i#7’ irreversible node, and the ‘ss-100’ deadlock node. The lack of incoming transitions to the rooted state shows unique conditions from which a modelled system is able to recover, but requires external, un-modelled influences/regulation to achieve.

It is possible to couple a perturbation with a defined initial state to fine-tune the behaviours of the system in response to known restrictions, or predict outcomes of new restrictions. In Fig. 2f, we see an STG generated when the initial state was defined as 111 with the P1 ectopic activity perturbation. The STG shows the routes available to the system using the logical parameters from Table 1 under the ectopic expression of P1, both of which lead to the deadlock state 100.

### Software

Logical modelling, network and perturbation analyses were performed using GINsim v3.0 [20]. The system verification was done using SMBioNet v3.1 [22]. Cytoscape [23] was used to visualise the networks.

## Results

### The regulatory network of cellular metabolic flexibility

In a review done previously [5], we had explored the pathways involved with cellular metabolic flexibility and had constructed a network of these pathways representing cellular metabolic flexibility. This network links both glucose and fatty acid oxidation with the TCA cycle as energy production methods, along with fatty acid (re)synthesis as an energy production and/or storage method. As mentioned previously in the Introduction section, metabolic flexibility at the cellular level enforces that only glucose or fatty acids are utilised for energy production at any given time. Exceptions to this enforcement have only been observed in situations involving high cellular stress and depleted nutrient conditions, such as in ischemic hearts [1].

**Fig. 3:**
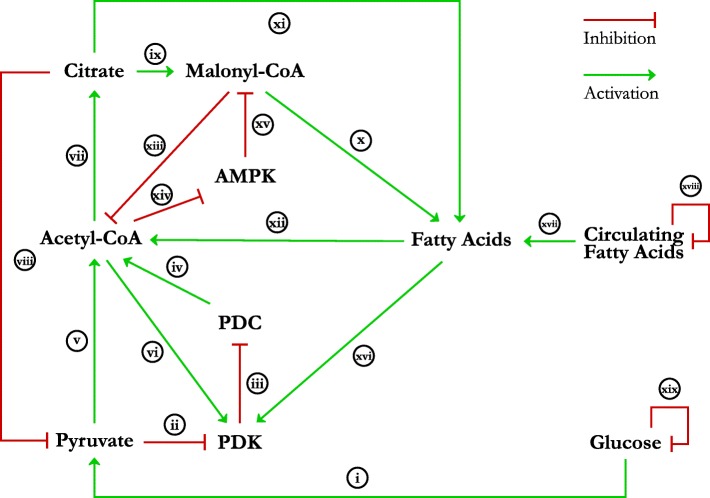
Biological regulatory network of cellular metabolic flexibility. The regulatory network consists of ten entities representing the biological processes involved in cellular metabolic flexibility. The entities interact with one another through various processes, abstractly represented here as activation or inhibition interactions. The interactions are labelled with Roman numerals, and are explained in Table 2

Figure 3 illustrates the complete biological regulatory network model. We have abstracted the larger cellular network of metabolic flexibility from the review to reduce the number of entities and thus reduce the complexity introduced by state-space explosion. Our regulatory network consists of ten entities, namely Glucose, Pyruvate, Pyruvate Dehydrogenase Kinase (PDK), Pyruvate Dehydrogenase Complex (PDC), Acetyl Coenzyme A (Acetyl-CoA), Citrate, Malonyl Coenzyme A (Malonyl-CoA), circulating fatty acids, cellular fatty acids (Fatty Acids), and adenosine monophosphate-activated protein kinase (AMPK). The interactions and processes represented by the edges of the biological network are explained in Table 2, and we direct the readers to the original review article [5] for the detailed explanation of the biological pathways and interactions involved in metabolic flexibility. Additional file 2 also shows a generic procedure for abstraction/reduction of regulatory networks. For a more detailed explanation of the reduction algorithm including the formal definitions and proofs, we refer the reader to Saadatpour et al. [24].

**Table 2:** Edge list and explanation of the interactions in biological regulatory network in Fig. 3

Edge Label	Interaction Explanation
i	Represents the process of glucose uptake and its multi-step conversion via various enzymes to Pyruvate [37, 38]
ii	Represents the allosteric inhibition of the PDK enzymes by Pyruvate [25].
iii	Represents the inhibition of PDC by PDKs via site-specific phosphorylation [1, 25].
iv	Represents the involvement of PDC in converting Pyruvate into Acetyl-CoA via decarboxylation [37, 38].
v	Represents the consumption of Pyruvate to create Acetyl-CoA via PDC mediated decarboxylation [26].
vi	Represents the allosteric activation of PDKs via NADH and ATP produced during the TCA cycle fuelled by Acetyl-CoA [1].
vii	Represents the conversion of Acetyl-CoA to Citrate in the mitochondria, part of which is transported into the cytoplasm [27, 39].
viii	Represents the inhibition of phosphofructokinases (PFKs) by cellular Citrate, thereby inhibiting the production of Pyruvate from Glucose [28, 29].
ix	Represents the conversion of Citrate to Malonyl-CoA through the Acetyl-CoA carboxylase 1 (ACACA) mediated carboxylation [1].
x	Represents the utilisation of Malonyl-CoA for fatty acid synthesis [27, 39].
xi	Represents the reconversion of Citrate to Acetyl-CoA in the cytoplasm to be used for fatty acid synthesis alongside Malonyl-CoA [27, 39].
xii	Represents the breakdown of fatty acids to Acyl-CoA, transport into the mitochondria via the carnitine transport process and conversion to Acetyl-CoA for the TCA cycle [30, 40].
xiii	Represents the inhibition of the carnitine transport process by Malonyl-CoA, thereby affecting Acetyl-CoA production [1].
xiv	Represents the negative effect of Acetyl-CoA on AMPK activity via higher ATP and lower AMP concentrations [1, 31].
xv	Represents the inhibition of Malonyl-CoA production by the AMPK mediated inhibition of ACACA [1, 31].
xvi	Represents the increased activity of PDKs by cellular fatty acids via Peroxisome Proliferator-Activated Receptor gamma (PPAR *γ*) signalling [25, 32–34].
xvii	Represents the uptake of circulating fatty acids into the cell [35, 36].
xviii	Highly abstracted representation of circulating fatty acid regulation outside the cell.
xix	Highly abstracted representation of circulating glucose regulation outside the cell.

### System verification of the logical parameters governing cellular metabolic flexibility

T h e selection of the logical p a r a m e t e r s f o r t h e r e g u l a t o r y n e t w o r k was done manually as most of the interactions present in the system are well-studied biological processes. The exception was the regulation of PDK as it is relatively less known and does not rely on stringent biochemical reactions, allowing for multiple regulatory possibilities. What is known is the inhibition of PDK isoenzymes via pyruvate, and the activation of PDK isoenzymes through either TCA cycle products or peroxisome proliferator-activated receptor gamma (PPAR *γ*) triggered by fatty acids in the cytoplasm [5]. Retaining these interactions, we get six combinations of logical parameters. We selected four sets of logical parameters for PDK, generating four models of our regulatory network (the remaining to two sets of logical parameters lead to either no effect of inhibition, or no effects of activation). These models are,

1. The inhibitor, Pyruvate, always blocks PDK activity when it is present in the system.
2. The inhibitor, Pyruvate, only blocks PDK activity when at least one of its activators, Acetyl-CoA (mediating activation via increased BADH and ATP) or Fatty Acids (mediating activation via PPAR *γ*), is absent from the system. Thus, the activators collectively override Pyruvate mediated inhibition.
3. Acetyl-CoA mediated activation of PDK bypasses Pyruvate mediated inhibition.
4. Fatty Acid mediated activation of PDK bypasses Pyruvate mediated inhibition.

**Fig. 4:**
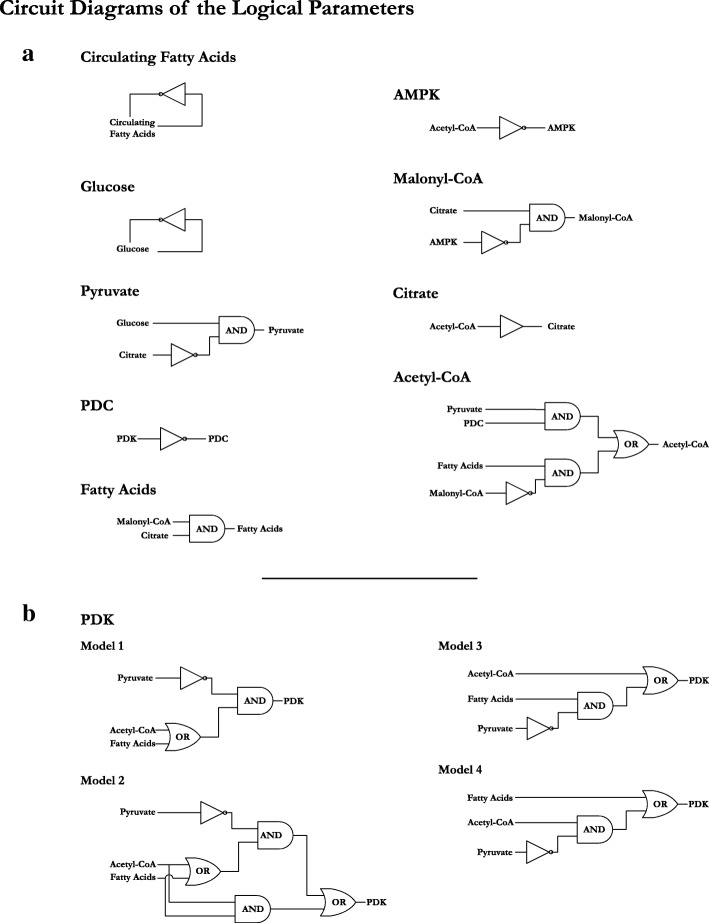
Circuit diagrams of the logical parameters for the regulatory network of cellular metabolic flexibility. **a**) The circuit diagrams representing the entities other than PDK. Each entity has a single circuit diagram representing the respective set of parameters. **b**) Shows the four models of PDK regulation, differing on how the activators (Fatty Acids and Acetyl-CoA) are able to affect the activation of PDK in the presence of the inhibitor (Pyruvate). The tabulated logical parameters for all entities are provided as Additional file 3
- Fatty acid Oxidation: Ensuring that fatty acid oxidation to acetyl-CoA takes place in the absence of malonyl-CoA
- Known PDK interactions: Ensuring that PDK inhibits glucose oxidation, allowing fatty acid oxidation to take place
- Absence of PDK: Ensuring glucose oxidation resumes in the absence of PDK, creating malonyl-CoA

Figure 4 shows the circuit diagram representations of the logical parameters for each entity, including the circuit diagrams for the four PDK parameter sets. The tabulated logical parameters are provided as Additional file 3. The four models were then tested in SMBioNet [22] for system verification and parameter selection. The following biological properties were tested,

- Glucose Oxidation: Ensuring that glucose is oxidised to pyruvate, which contributes to acetyl-CoA production

These biological properties are codified as CTL formulae in Table 3, and their formulation is explained in Additional file 4. All four models of PDK regulation were able to satisfy these properties, implying that the four logical parameter sets modelled for PDK are biologically plausible. The SMBioNet source file for the system verification of the regulatory network is provided in Additional file 5.

**Table 3:** CTL formulae used for system verification of the regulatory network of cellular metabolic flexibility

Property	CTL Formula
Glucose Oxidation	((*Glucose*=1∧*Pyruvate*=0∧*Citrate*=0)→*EX*(*Glucose*=1∧*Pyruvate*=1))
	∧
	((*Pyruvate*=1∧*PDC*=1∧*Acetyl*- *CoA*=0)→*EX*(*Pyruvate*=1∧*PDC*=1∧*Acetyl*- *CoA*=1))
Fatty acid Oxidation	((*Fatty* *Acids*=1∧*Malonyl*- *CoA*=0∧*Acetyl*- *CoA*=0)→
	*EX*(*Fatty* *Acids*=1∧*Malonyl*- *CoA*=0∧*Acetyl*- *CoA*=1))
Presence of PDK	((*PDK*=1∧*PDC*=1∧*Pyruvate*=1∧(*Fatty* *Acids*=0 | *Malonyl*- *CoA*=1)∧*Acetyl*- *CoA*=1)→
	*EF*(*PDK*=1∧*PDC*=0∧(*Fatty* *Acids*=0 | *Malonyl*- *CoA*=1)∧*Acetyl*- *CoA*=0))
	∧
	((*PDK*=1∧(*PDC*=0 | *Pyruvate*=0)∧*Fatty* *Acids*=1∧*Malonyl*- *CoA*=0∧*Acetyl*- *CoA*=0)→
	*EX*(*PDK*=1∧(*PDC*=0 | *Pyruvate*=0)∧*Fatty* *Acids*=1∧*Malonyl*- *CoA*=0∧*Acetyl*- *CoA*=1))
Absence of PDK	((*PDK*=0∧*PDC*=1∧*Pyruvate*=1∧(*Fatty* *Acids*=0 | *Malonyl*- *CoA*=1)∧*Acetyl*- *CoA*=0)→
	*EX*(*PDK*=0∧*PDC*=1∧*Pyruvate*=1∧(*Fatty* *Acids*=0 | *Malonyl*- *CoA*=1)∧*Acetyl*- *CoA*=1))
	∧
	((*PDK*=0∧*PDC*=1∧*Pyruvate*=1∧*Malonyl*- *CoA*=0∧*Acetyl*- *CoA*=1)→
	*EF*(*PDK*=0∧*PDC*=1∧*Pyruvate*=1∧*Malonyl*- *CoA*=1∧*Acetyl*- *CoA*=1))

### Network analysis of the behaviours exhibited by the models

Since all four parameter sets of PDK regulation passed system verification on known biological observations, we analysed all four models. The STG (state transition graph; see Methods section) of each model consisted of 1,024 states, and 6,144 transitions between the edges. The only difference between the STGs of the four models were the transitions between the states governing the regulation of PDK, which was expected as per the logical parameters. The STG of model 1 is provided in Additional file 6, and shows the size, density, and complexity of the network and behaviours contained within.

**Fig. 5:**
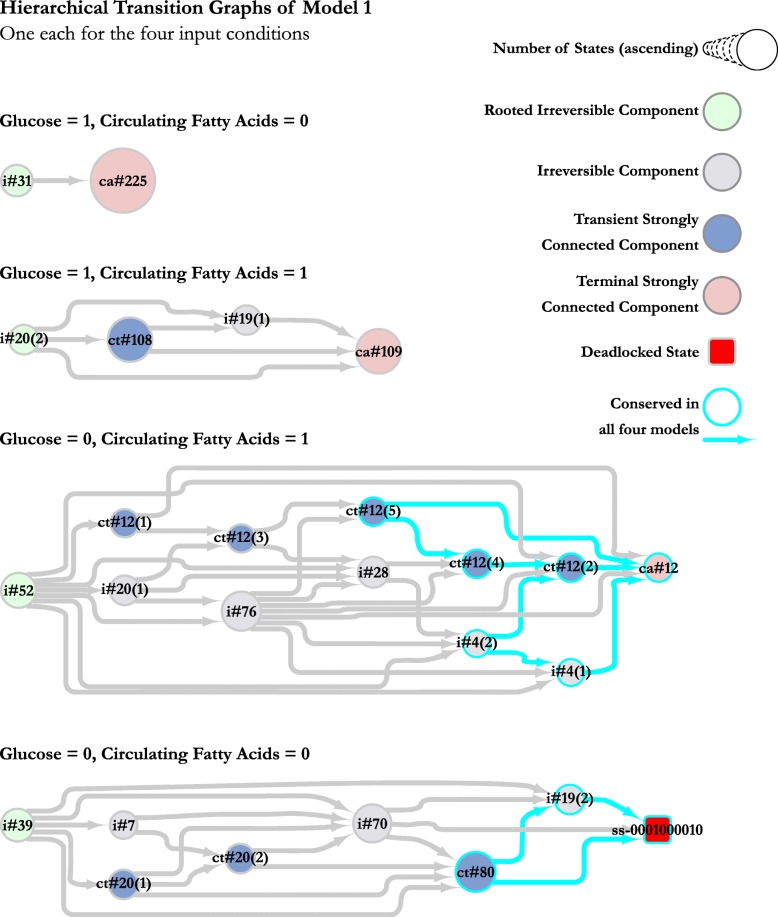
Hierarchical transition graphs (HTGs) of Model 1. Each node is labelled with a set of letters denoting the type of the node, followed by the number of states that node is representing. For states having the same type and number of states, a number in parentheses is added to the name to differentiate them. The size of the node represents the number of states contained within the node. The irreversible components (‘i#’) represent states which do not contain any cycles or homoeostatic behaviours. The strongly connected components (‘ct#’ and ‘ca#’) represent cyclic or homoeostatic behaviours. The deadlocked state (‘ss-’) represents a single state where the system dynamics seize to function. The nodes and edges in cyan represent the nodes and edges which are conserved in all four models of PDK regulation. The HTGs of the remaining models 2, 3 and 4 are provided as Additional file 7

We then proceeded to collapse the four STGs into their respective HTGs (hierarchical transition graphs; see Methods section), to compare the behavioural patterns and substructures in the models. We observed that due to the regulation of Glucose and Circulating Fatty Acids as inhibitory self-loops, the majority of the states from the STGs, 992 to be exact, are collapsed into a single node in the HTG with the remaining nodes showing fluctuations of Glucose and Circulating Fatty Acids without any effect on the cellular environment. Essentially, the various groups of system dynamics were being integrated together by the fluctuation of the glucose and circulating fatty acid input nodes. The self-inhibitory loop on each of the input nodes would switch them between 0 and 1, thereby linking all different strongly connected components together to form a single strongly connected component. Although this behaviour does show how interconnected the biological behaviours are, it makes the analysis of these behaviours that much complex. To remedy this situation, we opted to remove the self-inhibitory loops and restricted the input nodes to the four combinations of 0 and 1 to see how the system behaves for these particular input conditions. The only difference is that the edges that connected the pairs of states differing in only the level of either glucose or circulating fatty acids are no longer connected due to the absence of the self-inhibitory loop governing the change in level, neatly dividing the previously large 1,024 state STG into four smaller 256 state STGs for detailed analysis. We then proceeded with comparing the HTGs of each model with the respective input combination discussed below. Figure 5 shows the HTGs of Model 1 for all four input combinations.

#### Input 1: glucose only

This input combination models the availability of circulating glucose and the unavailability of circulating fatty acids. All four models of PDK regulation collapse the STG into a two node HTG containing all 256 states pertaining to this input combination. The root node is an irreversible component that does not contain any incoming edges, and consists of either 31, 33, 29 or 39 states for models 1, 2, 3 and 4 respectively. This root node shows a situation from which the system is able to recover to stable behaviours, but is unable to return to the original situation (hence the irreversible component). The remaining states are collapsed into the leaf node, which represents a terminal SCC. The system can remain in the terminal SCC indefinitely, following the cyclic behaviours it represents. In case the input conditions change, the system would then be able to move to complementary state in one of the HTGs representing the new input combination.

#### Input 2: both glucose and circulating fatty acids

In this input combination, both glucose and fatty acids are available for cellular metabolism. Models 1, 2 and 3 generate a four node HTG each, all consisting of two irreversible components (one of which is a root node), and two SCCs (one of which is a terminal SCC). Model 4 generates ten nodes, consisting of five irreversible components and five SCCs. We observed that the smaller HTGs of the first three models represent very similar sets of dynamics because the logical parameters for these models tie the regulation of PDK completely or partially to the TCA cycle. In model 4, this dependency is nullified as the Fatty Acid mediated activation via PPAR *γ* signalling is able to bypass the TCA cycle. This results in stable cyclic behaviours in the terminal SCC untying from each other and dispersing into smaller groups of cyclic behaviours, accompanied by irreversible components. However, the non-terminal SCC remains the same as those of models 1, 2 and 3. In essence, the larger structure of the HTGs remains intact.

#### Input 3: circulating fatty acids only

In this input combination, only circulating fatty acids are available for metabolism. All four models show strong divergence in their respective HTGs, with different types, number, and sizes of nodes. The only conserved pattern in the HTGs is the terminal SCC, along with three more non-terminal SCCs leading to the terminal SCC. This sub-network maintains both the number of states, as well as the edges between the respective SCCs across all four models, suggesting that although the change in PDK regulation has a strong effect in the upstream behaviours in the HTGs, the system converges to the same behaviours and patterns.

#### Input 4: no glucose or circulating fatty acids

This input combination represents an extreme scenario where neither circulating glucose nor circulating fatty acids are available to fuel metabolism. As such, the system moves towards cell death, which can be seen as the only deadlocked state (also known as stable state) in the all four HTGs, labelled as ‘ss-0001000010’. Models 2, 3 and 4 show very similar behaviour patterns in terms of the node types and the edges between them, whereas model 1 shows some behaviours as separate nodes instead. However, as with the previous input combination, this one also has a sub-network conserved between all four HTGs consisting of an SCC, an irreversible component, and the deadlocked state.

### Perturbation of regulatory components for impact propagation

In the perturbation analysis, we opted to perturb the non-metabolite inhibitors of the regulatory network to either ectopic or knockout levels to observe how their effects propagate through the system. The reason for selecting non-metabolite inhibitors is that the metabolite inhibitors are derived from the metabolic processes themselves, thus perturbing those would create a self-fulfilling prophecy in terms of gauging the effects of the regulators on cellular metabolic flexibility. The only two entities in our regulatory network fulfilling this criterion are PDK and AMPK.

We started with perturbing PDK to knockout level by restricting it to level 0. We then initialised the system with all entities at level 0 and tested it with all four input combinations. We observed the same behaviours as those in the network analysis for all input combinations, except for when both circulating glucose and circulating fatty acids are available for metabolism. For this input combination, we observed that when PDK is locked to level 0 the STG shows that the Acetyl-CoA is derivable from both glucose and fatty acid sources simultaneously, contrary to the known biological properties which were also checked via CTL model checking. When we allowed the system to change the PDK level to 1, we immediately observe only fatty acid driven Acetyl-CoA production.

We then proceeded to test PDK at ectopic level by restricting it to level 1 and testing again with the four input combinations (while the remaining entities of the system are initialised at level 0). We observed that, like previously, the behaviours were similar to those observed in the network analysis except for one input combination, this time it being the availability of circulating glucose and the absence of circulating fatty acids. The STG of this combination showed that there was no Acetyl-CoA production as the ectopic activity of PDK was barring Pyruvate conversion to Acetyl-CoA, and there were no available fatty acids to fuel metabolism. When we allowed the system to change the PDK level to 0, glucose driven metabolism resumed.

Lastly, we perturbed AMPK using the same method used for PDK. We did not observe any changes when AMPK was restricted to level 0. However, when restricted to level 1, we observed that changes in the behaviours generated when both the circulating glucose and circulating fatty acids are available. For this input combination, the ectopic activity of AMPK led to a fatty acid preferred metabolism, which, in turn, led to ectopic PDK levels, leading solely to fatty acid driven metabolism, instead of any switching behaviour. Here, we observed that AMPK perturbation still acted through PDK mediated regulation.

## Discussion

In this study, we have utilised a logical modelling and network analysis workflow to assess the hypothesis of the PDC-PDK regulatory switch being a key regulatory mechanism behind cellular metabolic flexibility. We start with the construction of the regulatory network by abstracting the cellular network of metabolic flexibility reviewed earlier f. The regulatory network, consisting of ten entities and nineteen interactions, covers both the glucose and fatty acid oxidative metabolism pathways, merging them with the fatty acid synthesis pathway along with AMPK and PPAR *γ* signalling pathways. The selection of the logical parameters for all but one of the entities was relatively straightforward as the biological processes being represented have been well studied [37–40]. The remaining entity, PDK, had four possible sets of logical parameters as it has competing activators and inhibitors in the regulatory network. As opposed to other entities, the competitive regulation of PDK is difficult to decipher because the interactions are not direct outputs of biochemical reactions. On top of this, various regulators of PDK have different intensities of regulations for the four PDK isoenzymes in different tissues [41].

The interactions in the regulatory network are both simplified and abstracted so we relied on system verification using model checking to test four possible logical parameter sets for PDK regulation. CTL formulae were used to formulate both oxidative metabolisms and the known behaviours of the PDC-PDK regulatory interactions. The model checker, SMBioNet [22] verified all four logical parameter sets of PDK to contain the formulated known behaviours, leading us to proceed further with four models differing on the regulation of PDK. The verification of all four logical parameter sets shows the biological plausibility of the four different types of PDK regulation, in line with the multiple intensities and tissue specific regulation discussed in [41]. In addition, the various STGs and HTGs generated by the four models show that the system eventually settled into very similar dynamics for the different sets of inputs, again supporting the biological plausibility of the four models. The STGs and HTGs differed only in the upstream regulation of the dynamics because of the difference in PDK regulation. However, the simplicity of the abstraction used in our regulatory network, both in terms of representing the four PDK isoenzymes as a single entity as well as merging the various regulations (such as acetyl-CoA, NADH and ATP mediated activation of various PDKs into a single edge), limits our model to being non-tissue specific. This limitation also affects the elucidation of the contexts involved and/or required by the four models to exhibit the behaviours presented in this study. As the base model itself is not completely tissue specific, it is possible that these four models broadly represent different paths of metabolic flexibility in different tissues. However, it is equally likely that these four models can exist in the same tissue but at different times because of other effects not modelled in this study (such as epigenetic effects over time).

As an additional verification step, we constructed the larger cellular metabolic flexibility network from our review [5] in the software GINsim, consisting of 63 entities and 81 edges. This network included one modification from the network depicted in our review – two inhibitory edges, one from ATP to AMP and the other from NADH to NAD+, were added to account for their cycling in the cell. We then proceeded to find the deadlocked states in the larger network to compare with the network we had manually constructed in this study. We found that the larger network generated only one deadlocked state. However, comparing only the ten entities modelled in the network in this study, we found that the levels of these ten entities were the same for both deadlocked states. We also used the reduction tool offered in GINsim that computationally reduces a model. We reduced the larger network, deselecting the ten entities found in our manually constructed model in this study, making the reduction algorithm preserve them. The reduced model generated by GINsim consisted of eleven entities, with the additional entity being the node for TCA cycle. This reduced model also generated a single deadlocked state, as was expected because the reduction algorithm preserves the mathematical constraints in the model. This deadlocked state only showed two entities deadlocked at level 1, PDC and AMPK, the same as the ones from our manually constructed model (shown in the “Input 4:” sub-sub-section of the Results section). These comparisons serve as an alternative verification method for the manual construction of the network presented in this study. The additional models are provided as Additional file 8.

The perturbation analysis, done by restricting certain components to knockout or ectopic expressions, allowed us to test the propagation of regulation in the regulatory network when the negative regulators in the system malfunctioned. We elected to perturb the non-metabolite negative regulators, namely PDK and AMPK, because we wanted to test the regulation of the switching of metabolism from glucose to fatty acids independent of the increase or decrease in metabolite concentrations implied in logical modelling. The results of the perturbation analysis reveal that malfunctioning of PDK has a direct effect on the switching of metabolism, which is in line with our hypothesis. What is more interesting is that the perturbation of the only other non-metabolite inhibitor modelled in our regulatory network, AMPK, also propagated its effects through persistent activation or availability of PDK, providing additional support to our hypothesis. The results of the perturbation analysis, coupled with the conserved dynamics in all four models reinforces the hypothesis that the PDK isoenzymes are a key regulatory element of cellular metabolic flexibility via the PDC-PDK interaction.

This propagation of the regulatory effect places the PDK enzymes squarely in the middle of perturbed metabolism, as can be seen in cancer studies [42, 43] where PDKs were found to be over-expressed. The reprogramming of cellular metabolism has been identified as a new hallmark of cancer where the cellular metabolism of the cancerous cells moves away from complete glucose oxidation to just glycolysis [44]. In the aforementioned cancer studies, PDK expression was suppressed via treatment with Dichloroacetate (DCA), an inexpensive small molecule suppressor, to switch metabolism over to glucose oxidation. Similar treatments in other studies has shown that the DCA treatment caused apoptosis in cancer cells [45–48]. However, on the other side of the spectrum, a study targeting Alzheimer’s disease via rat central nervous system cell line models found that the overexpression of PDK1 (along with lactate dehydrogenase A) conferred a resistance to Ameloid *β* and other toxins, thereby mitigating some of the mechanisms underlying Alzheimer’s disease progression [49]. When taken collectively, these studies indicate that the balance of PDK enzyme expressions play an important role in the health of various cell types, thus, relying on the cellular metabolic flexibility through the PDC-PDK regulatory interaction. In addition, this maintenance of cellular metabolic flexibility as well as the tweaking of the PDC-PDK switch can be further used to supply new drug targets for the aforementioned ailments and conditions.

In cell types reliant on a single substrate for oxidative metabolism, the role of the metabolic switch is slightly different, likely to regulate the rate of oxidative metabolism instead of metabolic flexibility. One example of such cell types are Endothelial cells which utilise glucose. We took the network resource developed in our previous study [5] and visualised baseline RNA-Seq expression data for endothelial cells from the BLUEPRINT Epigenome project [50] (available from ArrayExpress as E-MTAB-3827). These endothelial cells were extracted from the umbilical vein during proliferating and resting states (the visualisation is provided as Additional file 9). We observed that the expression of PDKs is very low, and no expression of PPAR *γ* is taking place, indicating the absence of cellular fatty acid mediated PPAR *γ* signalling. Interestingly, there is expression of Stearoyl-CoA desaturase 1 (SCD), an enzyme from the fatty acid (re)synthesis pathway. However, studies have shown that SCD in endothelial cells plays a vital role in mitigating laminar stress [51, 52], thereby justifying its expression and indicating the limited role of the PDC-PDK regulatory switch in such cell types.

In essence, all four models of PDK regulation analysed in this study, coupled with the network and perturbation analyses, strongly suggest the PDK mediated inactivation of PDC as a key switching mechanism of cellular metabolism from glucose to fatty acids, and therefore, a key regulator of cellular metabolic flexibility. However, the model has its limitations, not least of which is that logical modelling is time and quantity independent. This means that that although our models suggests the PDC-PDK switch as a key regulator, it does not tell us anything about the intensity and duration of this regulation. In addition, wet-lab experimentation is still required to validate our findings, the design of which is difficult in itself since many of the interactions and regulations modelled in the system happen at extremely short time scales, the accurate measurement of which is tricky at best. These design constraints themselves are one of the reasons why quantitative data was sparse and lacking at the time of this study, resulting in the logical modelling conducted in this study. While our study focuses on the substrate switching in metabolism and specifically the enzymatic regulation of this substrate switching, there are other factors/mechanisms of the regulation of cellular metabolism that are not considered in our current model. One of these is the ratio of deuterium to hydrogen in the cellular environment, which has been shown to affect cellular metabolism [53, 54]. Although this aspect is out of the scope of the current study, it would be interesting to further investigate its impact on the regulation of cellular metabolism and metabolic flexibility in future studies.

## Conclusion

In this study, we have modelled and analysed cellular metabolic flexibility using logical modelling and network analysis. The results of our models strongly suggest that the PDC-PDK regulatory switch plays an important role in the regulation of cellular metabolic flexibility, revolving around the TCA cycle and the oxidative metabolism of glucose and fatty acids. The results support the hypothesis that this regulatory switch relies on the regulation of PDK itself, and thus PDK regulation acts as the pivot balancing cellular metabolic flexibility between available nutrients.

## Additional files

Additional file 1 Archive containing the SMBioNet and GINsim source files for toy example (ZIP 1.66 kb).

Additional file 2 PDF showing a generic abstraction/reduction process for regulatory networks (PDF 573 kb).

Additional file 3 Tabulated logical parameters for the regulatory network of cellular metabolic flexibility (PDF 60.1 kb).

Additional file 4 Explanation and conversion of the biological properties into computation tree logic (CTL) (PDF 97.5 kb).

Additional file 5 Archive containing the SMBioNet and GINsim source file for the regulatory network of cellular metabolic flexibility (ZIP 10.1 kb).

Additional file 6 Cytoscape session file of the STGs of Models 1, 2, 3 and 4 (CYS 967 kb).

Additional file 7 Cytoscape session file of the HTGs of Models 1, 2, 3 and 4 (CYS 82.4 kb).

Additional file 8 Archive containing alternative verification models from the review article constructed in GINsim (ZIP 11.3 kb).

Additional file 9 Cytoscape session file visualising the RNA-seq expression data of endothelial cells (CYS 58.6 kb).

## Data Availability

The Blueprint Consortium data visualised in this study (Additional File 7) is available from ArrayExpress as E-MTAB-3827 [https://www.ebi.ac.uk/arrayexpress/experiments/E-MTAB-3827/].
