# Supplementary material for: Logical modelling reveals the PDC-PDK interaction as the regulatory switch driving metabolic flexibility at the cellular level
Source: Genes Nutr. 2019 Sep 9;14:27. doi: 10.1186/s12263-019-0647-5 (PMC6734263; doi:10.1186/s12263-019-0647-5)

# Network Abstraction

- Problem/scenario specific

- Linear processes
  - A → B → C = A → C
  - A ⊣ B ⊣ C = A → C
  - A → B ⊣ C = A ⊣ C

- Branched processes
  - Require preserving the behaviour

- Reduces complexity for computation (state-space explosion) and analyses

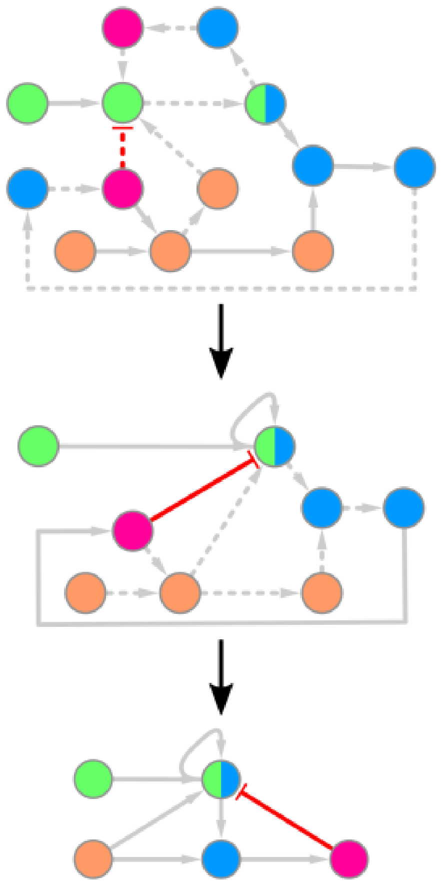

Supplement: Supplementary file 2 — PDF showing a generic abstraction/reduction process for regulatory networks (PDF 573 kb). [file 12263_2019_647_MOESM2_ESM.pdf]
