# Supplementary material for: Logical modelling reveals the PDC-PDK interaction as the regulatory switch driving metabolic flexibility at the cellular level
Source: Genes Nutr. 2019 Sep 9;14:27. doi: 10.1186/s12263-019-0647-5 (PMC6734263; doi:10.1186/s12263-019-0647-5)
Supplement: Supplementary file 3 — Tabulated logical parameters for the regulatory network of cellular metabolic flexibility (PDF 60.1 kb). [file 12263_2019_647_MOESM3_ESM.pdf]

Table 1: **Logical parameters of the regulatory network of cellular metabolic flexibility. The format used here represents the presence of respective entities in the system when they are listed in the parameter set. For PDK, there are four distinct models, the parameter values for which are listed in order, separated by commas.**

Entity	Parameter Set	Target Value			
Glucose	{ }	1			
	{Glucose}	0			
Circ-FA	{ }	1			
	{Circ-FA}	0			
Pyruvate	{ }	0			
	{Citrate}	0			
	{Glucose}	1			
	{Citrate, Glucose}	0			
PDC	{ }	1			
	{PDK}	0			
Acetyl-CoA	{ }	0			
	{PDC}	0			
	{Pyruvate}	0			
	{Malonyl-CoA}	0			
	{Fatty Acids}	1			
	{PDC, Pyruvate}	1			
	{PDC, Malonyl-CoA}	0			
	{PDC, Fatty Acids}	1			
	{Pyruvate, Malonyl-CoA}	0			
	{Pyruvate, Fatty Acids}	1			
	{Malonyl-CoA, Fatty Acids}	0			
	{PDC, Pyruvate, Malonyl-CoA}	1			
	{PDC, Pyruvate, Fatty Acids}	1			
	{PDC, Malonyl-CoA, Fatty Acids}	0			
	{Pyruvate, Malonyl-CoA, Fatty Acids}	0			
	{PDC, Pyruvate, Malonyl-CoA, Fatty Acids}	1			
Citrate	{ }	0			
	{Acetyl-CoA}	1			
AMPK	{ }	1			
	{Acetyl-CoA}	0			
Malonyl-CoA	{ }	0			
	{AMPK}	0			
	{Citrate}	1			
	{AMPK, Citrate}	0			
Fatty Acids	{ }	0			
	{Circ-FA}	1			
	{Citrate}	0			
	{Malonyl-CoA}	0			
	{Circ-FA, Citrate}	1			
	{Circ-FA, Malonyl-CoA}	1			
	{Citrate, Malonyl-CoA}	1			
	{Circ-FA, Citrate, Malonyl-CoA}	1			
		Model 1	Model 2	Model 3	Model 4
PDK	{ }	0	0	0	0
	{Pyruvate}	0	0	0	0
	{Fatty Acids}	1	1	1	1
	{Acetyl-CoA}	1	1	1	1
	{Pyruvate, Fatty Acids}	0	0	0	1
	{Pyruvate, Acetyl-CoA}	0	0	1	0
	{Fatty Acids, Acetyl-CoA}	1	1	1	1
	{Pyruvate, Fatty Acids, Acetyl-CoA}	0	1	1	1
