# Supplementary material for: Logical modelling reveals the PDC-PDK interaction as the regulatory switch driving metabolic flexibility at the cellular level
Source: Genes Nutr. 2019 Sep 9;14:27. doi: 10.1186/s12263-019-0647-5 (PMC6734263; doi:10.1186/s12263-019-0647-5)
Supplement: Supplementary file 4 — Explanation and conversion of the biological properties into computation tree logic (CTL) (PDF 97.5 kb). [file 12263_2019_647_MOESM4_ESM.pdf]

Explanation and conversion of the biological properties into computation tree logic (CTL)

Samar H.K. Tareen[1,*], Martina Kutmon[1,2], Ilja C. W. Arts[1,3], Theo M. de Kok[1,4], Chris T. Evelo[1,2], Michiel E. Adriaens[1]

December 3, 2018

1 Glucose Oxidation

In glucose oxidation in human cells, glucose undergoes glycolysis to form pyruvate, and is then converted into acetyl-CoA through decarboxylation via the pyruvate dehydrogenase complex (PDC). The glycolysis step can be inhibited by citrate in the cytoplasm. This two-step process is represented as a conjunction of two formulae in the computation tree logic (CTL),

- Glucose undergoes glycolysis to form pyruvate, which can be inhibited by cellular citrate. In a state where glucose and pyruvate are present, while citrate is not, at least one successive state should exist where glucose should be able to produce pyruvate,
  $((Glucose = 1 \wedge Pyruvate = 0 \wedge Citrate = 0) \rightarrow EX(Glucose = 1 \wedge Pyruvate = 1))$
- In a state where pyruvate becomes available in the presence of PDC, it is converted into acetyl-CoA in at least one successive state,
  $((Pyruvate = 1 \wedge PDC = 1 \wedge Acetyl\text{-}CoA = 0) \rightarrow EX(Pyruvate = 1 \wedge PDC = 1 \wedge Acetyl\text{-}CoA = 1))$

2 Fatty Acid Oxidation

Fatty acid oxidation encompasses their degradation into fatty acyl-CoA which are then transported into the mitochondria through the carnitine transport process by carnitine palmitoyltransferases (CPTs). Cellular malonyl-CoA, however, is responsible for targetting the CPTs and blocking this uptake. This process is represented in CTL as,

- In a state where fatty acids are available and malonyl-CoA is not, there exists at least one successor state where fatty acids produce acetyl-CoA,
  $((Fatty\,Acids = 1 \wedge Malonyl\text{-}CoA = 0 \wedge Acetyl\text{-}CoA = 0) \rightarrow$
  $EX(Fatty\,Acids = 1 \wedge Malonyl\text{-}CoA = 0 \wedge Acetyl\text{-}CoA = 1))$

3 Presence of PDK

In cellular metabolism, pyruvate dehydrogenase kinase (PDK) isoenzymes regulate the activity of PDC by inhibition through site specific phosphorylation. This action results in the unavailability of PDCs to convert pyruvate to acetyl-CoA. Thus cellular metabolism shifts to fatty acid oxidation driven acetyl-CoA production, provided malonyl-CoA is unable to block fatty acyl-CoA uptake into the mitochondria. In CTL, these properties are exhibited as,

- In a state where PDK becomes available when acetyl-CoA is being produced in the presence of pyruvate and PDC, while either fatty acids are not available or malonyl-CoA is inhibiting the fatty acyl-CoA uptake, then acetyl-CoA production would eventually cease because of the inhibition of PDC,
  $((PDK = 1 \wedge PDC = 1 \wedge Pyruvate = 1 \wedge (Fatty\,Acids = 0 \mid Malonyl\text{-}CoA = 1) \wedge Acetyl\text{-}CoA = 1) \rightarrow$
  $EF(PDK = 1 \wedge PDC = 0 \wedge (Fatty\,Acids = 0 \mid Malonyl\text{-}CoA = 1) \wedge Acetyl\text{-}CoA = 0))$

- In a state where PDKs are active and glucose oxidation has ceased, provided that fatty acid are available and malonyl-CoA is not, then acetyl-CoA would be produced based on fatty acid oxidation,
  $((PDK = 1 \wedge (PDC = 0 \mid Pyruvate = 0) \wedge Fatty\,Acids = 1 \wedge Malonyl\text{-}CoA = 0 \wedge Acetyl\text{-}CoA = 0) \rightarrow$
  $EX(PDK = 1 \wedge (PDC = 0 \mid Pyruvate = 0) \wedge Fatty\,Acids = 1 \wedge Malonyl\text{-}CoA = 0 \wedge Acetyl\text{-}CoA = 1))$

4 Absence of PDK

When PDK isoenzymes are not available, then their inhibitory effect on PDC ceases, allowing PDC to convert pyruvate into acetyl-CoA, provided glucose is available to be oxidised. This allowance contributes to the production of acetyl-CoA, parallel to fatty acid oxidation. Over production leads to citrate escaping to the cytoplasm and reducing glucose oxidation, but more importantly, citrate converted into malonyl-CoA which blocks uptake of fatty acyl-CoA, thereby switching cellular metabolism to glucose oxidation. In CTL, these properties are exhibited as,

- In a state where PDK isoenzymes are unavailable, pyruvate and PDC are available, and fatty acids either not available or malonyl-CoA blocking fatty acyl-CoA uptake, acetyl-CoA will be produced by the decarboxylation of pyruvate by PDC in a successive state,
  $((PDK = 0 \wedge PDC = 1 \wedge Pyruvate = 1 \wedge (Fatty\,Acids = 0 \mid Malonyl\text{-}CoA = 1) \wedge Acetyl\text{-}CoA = 0) \rightarrow$
  $EX(PDK = 0 \wedge PDC = 1 \wedge Pyruvate = 1 \wedge (Fatty\,Acids = 0 \mid Malonyl\text{-}CoA = 1) \wedge Acetyl\text{-}CoA = 1))$

- In a state where the absence of PDK has allowed acetyl-CoA production through glucose oxidation to proceed, an eventual state is reached where malonyl-CoA is produced, thus blocking fatty acid oxidation, $((PDK = 0 \wedge PDC = 1 \wedge Pyruvate = 1 \wedge Malonyl\text{-}CoA = 0 \wedge Acetyl\text{-}CoA = 1) \rightarrow$ $EF(PDK = 0 \wedge PDC = 1 \wedge Pyruvate = 1 \wedge Malonyl\text{-}CoA = 1 \wedge Acetyl\text{-}CoA = 1))$

References

[1] Tareen SHK, Kutmon M, Adriaens ME, Mariman ECM, de Kok TM, Arts ICW, et al. Exploring the cellular network of metabolic flexibility in the adipose tissue. Genes & Nutrition. 2018;13(1):17. doi:10.1186/s12263-018-0609-3.
